# Deconstructing the trip treatment: are hallucinogenic effects critical to the therapeutic benefits of psychedelics?

**DOI:** 10.1038/s44277-025-00043-y

**Published:** 2025-08-20

**Authors:** Albert Garcia-Romeu

**Affiliations:** Center for Psychedelic and Consciousness Research, Department of Psychiatry and Behavioral Sciences, Johns Hopkins University School of Medicine, Baltimore, MD USA

**Keywords:** Drug development, Pharmaceutics

## What’s in a name?

Interest in “psychedelic therapies,” using substances such as ketamine, 3,4-methylenedioxymethamphetamine (MDMA), and psilocybin to treat psychiatric conditions, has grown steadily in recent years. Although pharmacologically distinct and functioning via differential mechanisms, these drugs are broadly classified as hallucinogenic, meaning they are thought to produce hallucinations. However, this is contentious as evidenced by the wide-ranging terminology used historically to describe them. In addition to ‘hallucinogens,’ early researchers proposed such drugs might variably be called psychotomimetics (mimicking psychosis), phantastica (enhancing fantasy and imagination), and psychedelics (mind-manifesting) [1], highlighting the profound, complex mind-altering effects these substances can elicit. From a technical standpoint, most hallucinogens rarely induce true hallucinations where individuals experience false perceptions they believe to be genuine, though perceptual alterations like pseudo-hallucinations, synaesthesia, and illusions are common (Table 1). Furthermore, changes in higher-order processes like thinking, emotions, sense of time, self, and bodily awareness are typical, and these subjective effects have more often been linked to therapeutic changes than hallucinations *per se*.

**Table 1:** Glossary of key terms.

Key Terms	Definitions
Classic Psychedelic	The term “psychedelic” was first coined by Dr. Humphrey Osmond in 1957 to describe the mind-altering effects of drugs that are thought to act primarily as agonists at the serotonin 2 A receptor (5-HT_2A_R) (e.g., psilocybin, mescaline, LSD, DMT, but not ketamine). The word has since become synonymous with countercultural art and music, and a nonspecific catch-all term for hallucinogenic and visionary experiences, hence the addition of ‘Classic’ to denote this subset of 5-HT_2A_R agonist hallucinogens.
Delusion	Delusions reflect changes in normal thinking processes, generally when a person can become very confident in spurious beliefs (e.g., people are plotting against them or they are receiving special messages from the world around them). In psychedelic experiences, changes in thinking may reflect paranoid or grandiose delusions that could be considered adverse events, though may also relate to experiences viewed as insightful or spiritual (e.g., ego-loss, or experiencing oneself as one with the universe).
Dissociative Anesthetic	A term first introduced in 1965 by Dr. Edward F. Domino and colleagues, coined by Domino’s wife Toni, to describe the dreamlike state induced by the drug ketamine. These drugs are primarily thought to act as glutamatergic NMDA-receptor antagonists.
Entactogen	A term first introduced by Dr. David E. Nichols in 1986 to describe the effects of MDMA and similar compounds that can amplify positive emotions, empathy, and social connectedness. These drugs are primarily though to act via serotonin, norepinephrine, and dopamine reuptake inhibition and release.
Hallucination	Hallucinations are false sense percepts without corresponding stimuli in the external world, which can exist across any sensory domain (i.e., vision, hearing, smell, taste, and touch / interoception). Elementary hallucinations comprise basic false perceptions like a taste of sweetness or a hum, whereas complex hallucinations can be far more elaborate like seeing entire landscapes of intricate scenery or hearing voices telling a story.
Hallucinogen	A pharmacologically diverse group of psychoactive drugs that can cause perceptual alterations (including hallucinations, pseudo-hallucinations, and illusions, defined below) and widely varied changes in thinking, emotion, and sense of self. As with all drugs, these effects may differ according to dose and route of administration.
Illusion	Illusions are defined as perceptual distortions or misinterpretations related to actual objects, such as seeing something as larger or smaller than it really is or perceiving a false sense of movement in stationary objects.
Ketamine	Ketamine is a dissociative anesthetic that is used widely as an anesthetic and at sub-anesthetic doses has also shown promise as a rapid-acting treatment for depression, suicidality, and substance use disorders (among others). Side effects may include drowsiness, nausea, disorientation, and increased blood pressure and heart rate.
MDMA	MDMA is an entactogen that has primarily been studied and shown effectiveness for the treatment of post-traumatic stress disorder (PTSD) in combination with structured therapy. Side effects may include increased blood pressure and heart rate, headache, and hyperthermia.
Pseudo-hallucination	Pseudo-hallucinations may constitute elements of both elementary and complex hallucinations, but with the accompanying insight that the perceptions are unreal or imagined, which is typically lacking in true hallucinations.
Psilocybin	Psilocybin is a naturally occurring classic psychedelic that has shown potential as a treatment for depression, anxiety, existential distress, and substance use disorders (among others), typically in combination with psychological support. Side effects may include increased blood pressure and heart rate, transient anxiety, panic or paranoia, nausea, and headache.
Psychonaut	Psychonaut refers to a person who endeavors to explore the mind or psyche, often via techniques such as ingesting psychoactive substances, meditation, or sensory deprivation.
Psychoplastogen	The term psychoplastogen was first coined by Dr. David E. Olson in 2018 to denote drugs that can promote rapid structural and functional neural plasticity such as growth of new synapses and dendrites between neurons. This psychoplastogenic property has been proposed as a biological mechanism underlying the rapid-acting therapeutic effects of various drugs including ketamine, MDMA, and psilocybin.
Synaesthesia	Synaesthesia is an intermingling of sensory signals when information from one sense elicits corresponding sensory experiences in another domain, for instance a sense of tasting colors, or seeing sounds.

## History

Humans have sought out hallucinogens like mescaline and psilocybin since prehistoric times. Early research in the 20th century, largely surrounding the semisynthetic ‘classic psychedelic’ lysergic acid diethylamide (LSD), sought to understand the biochemical basis of these drugs’ psychotropic effects and examined their therapeutic potential with mixed results [2]. More recently, novel research suggests these substances may hold substantial therapeutic potential across various conditions. Ketamine, first studied as an anesthetic, has since demonstrated rapid-acting antidepressant effects and promise for treating substance use disorders and other conditions. The entactogen MDMA has shown positive results with structured therapy for treating post-traumatic stress disorder (PTSD). Psilocybin, found in ‘magic mushrooms,’ has shown rapid-acting antidepressant and anxiolytic effects and potential for other conditions including substance use disorders. Even among experts, views on terminology and classification vary, but for this review, these substances are highlighted as good exemplars representing a growing body of contemporary research. Other hallucinogens currently under investigation include the kappa-opioid agonist salvinorin A, the serotonergic agonist 5-MeO-DMT, and the atypical psychedelic ibogaine, though fall outside the scope of this primer.

## Subjective effects and altered states of consciousness

In addition to growing evidence suggesting medical value, both scientists and psychonauts have held a long-standing appreciation for hallucinogens’ mind-altering effects. As Aldous Huxley noted in his 1954 essay on mescaline, *The Doors of Perception*, “To be shaken out of the ruts of ordinary perception, to be shown for a few timeless hours the outer and the inner world, not as they appear to an animal obsessed with survival or to a human being obsessed with words and notions, but as they are apprehended, directly and unconditionally, by Mind at Large— this is an experience of inestimable value to everyone and especially to the intellectual.” Since those early days, researchers observed the occurrence and seeming import of such experiences as possibly mediating therapeutic effects, leading some to propose the insights and realizations induced by hallucinogens can result in lasting behavioral changes and improvements in anxiety or depression. This question lies at the crux of the present discussion, namely, are hallucinogenic effects critical to the therapeutic benefits of psychedelics?

## Associations between subjective effects and therapeutic outcomes

Beyond the pharmacological mechanisms delineated here, an equally critical factor to consider is the particular subjective qualities of the drug experiences. Dissociative anesthetics like ketamine have been characterized as inducing out-of-body and dreamlike states. Entactogens like MDMA can produce heightened social connection, empathy, and positive emotions. Classic psychedelics like psilocybin and LSD can promote states described as ego dissolution, oceanic boundlessness, mystical-type experience, and emotional breakthroughs [3]. To date, the literature is mixed on whether subjective effects of ketamine and classic psychedelics are associated with therapeutic benefits [4]. However, meta-correlation of available data indicates that for ketamine, an estimated 5–10% of variance in therapeutic outcomes may be predicted by specific subjective effects such as a sense of unity, awe, or insight, meaning the degree to which people experience these under ketamine accounts for roughly this much improvement in symptoms like depression and substance misuse afterwards. For psilocybin, this is closer to 24% [5], congruent with recent psilocybin clinical trial data [6]. Notably, MDMA has not shown associations between acute subjective effects and persisting therapeutic benefits, though this has not been studied systematically and may reflect differences in mechanism or targeted conditions. Although these associations suggest some modest relationship between subjective effects and mental health improvements, it has been posited that such effects may be unnecessary and perhaps undesirable adverse effects [7]. Preclinical data implicates neuroplasticity-enhancing and anti-inflammatory effects of psychedelics as potential biological mechanisms driving therapeutic benefits [8]. Researchers are now developing and testing novel compounds to assess whether these biological effects can be leveraged to exert therapeutic efficacy in absence of hallucinogenic effects [9]. Animal data supports this as a promising path forward. As rigorous human testing progresses (now in early stages), we will learn more about these compounds’ subjective effects, biological impact, and therapeutic potential, helping elucidate the role of hallucinogenic effects in psychedelics’ (and related compounds’) therapeutic efficacy.

## The role of therapy and other factors

**Fig. 1:**
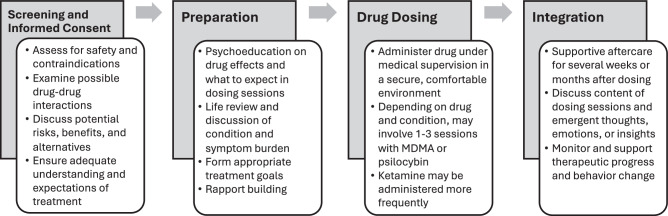
General Phases of Psychedelic Therapy.

Another ongoing debate in the field surrounds the necessity of structured therapy or psychological support in promoting therapeutic change. Are hallucinogenic experiences or psychedelics’ biological effects sufficient to produce significant symptom improvements, or do these simply serve as catalysts for therapeutic processes occurring between a patient and therapist? [10] It is generally agreed that some form of structured support, including psychoeducation on drug effects, review of life history and symptom burden, and formation of treatment goals can be helpful in facilitating the process (Fig. 1), serving as a bridge between the acute altered states experienced under the influence of psychedelics with longer-term altered traits targeted in therapy like reduced rumination, anxious preoccupation, or addictive behaviors [11]. The relative contribution of drug vs. psychological support remains to be determined, though preliminary data indicate the therapeutic relationship can impact treatment outcomes [12], and factors such as environment, personality, and mindset can influence subjective drug effects. These findings highlight the complex interplay between multiple elements involved in hallucinogenic effects and psychedelic therapies.

## Conclusion

In conclusion, investigation into hallucinogens, their subjective effects, biological underpinnings, risks, and therapeutic potentials is expanding rapidly. Many questions remain unanswered, and issues such as how to maintain blinding when studying drugs with pronounced psychoactive effects, and the contribution of expectancy and placebo effects to therapeutic outcomes present compelling challenges for the field. Nevertheless, the promise of psychedelic therapies in psychiatry and beyond has generated substantial enthusiasm from the scientific community and general public, warranting further research to inform their clinical utility and mechanisms. In tandem with modern neuroimaging and analytic methodologies, these drugs will likely yield novel insights into the nature of conscious experience and its relationship to neurochemistry and neurobiology.

### Citation diversity statement

The authors have attested that they made efforts to be mindful of diversity in selecting citations for this article.
